# Diagnostic Efficacy of Five Different Imaging Modalities in the Assessment of Women Recalled at Breast Screening—A Systematic Review and Meta-Analysis

**DOI:** 10.3390/cancers16203505

**Published:** 2024-10-17

**Authors:** Judith Akwo, Ibrahim Hadadi, Ernest Ekpo

**Affiliations:** 1 Medical Image Optimisation and Perception Group, Faculty of Medicine and Health, Discipline of Medical Imaging Science, The University of Sydney, Sydney, NSW 2050, Australia; 2 Department of Radiological Sciences, College of Applied Medical Sciences, King Khalid University, Abha 62529, Saudi Arabia; ihadadi@kku.edu.sa; 3 Department of Imaging and Radiation Therapy, Brookfield Health Sciences Complex, University College Cork, College Road, T12 AK54 Cork, Ireland

**Keywords:** breast cancer, breast assessment, diagnostic work-up, additional testing, imaging assessment

## Abstract

There are variations in the assessment pathways for women recalled at screening, and the imaging assessment pathway with the best diagnostic outcome is poorly understood. This paper examines the efficacy of five imaging modalities for the assessment of screen-recalled breast lesions. **Methods:** The Preferred Reporting Items for Systematic Reviews and Meta-analyses (PRISMA) strategy was employed to identify studies that assessed the efficacy of imaging modalities in the assessment of lesions recalled at screening from the following eight databases: Medline, Web of Science, Embase, Scopus, Science Direct, PubMed, CINAHL, and Global Health. Search terms included “Breast assessment” AND “Diagnostic Workup” OR “Mammography” AND “Digital Breast tomosynthesis” AND “contrast enhanced mammography and Magnetic Resonance imaging” AND “breast ultrasound”. Studies that examined the performance of digital mammography (DM), digital breast tomosynthesis (DBT), handheld ultrasound (HHUS), contrast-enhanced mammography (CEM), and magnetic resonance imaging (MRI) in screen-recalled lesions were reviewed. Meta-analyses of these studies were conducted using the MetaDisc 2.0 software package. **Results:** Fifty-four studies met the inclusion criteria and examined between one and three imaging modalities. Pooled results of each imaging modality demonstrated that CEM has the highest sensitivity (95; 95% CI: 90–97) followed by MRI (93; 95% CI: 88–96), DBT (91; 95% CI: 87–94), HHUS (90; 95% CI: 86–93), and DM (85; 95% CI: 78–90). The DBT demonstrated the highest specificity (85; 95% CI: 75–91) followed by DM (77; 95% CI: 66–85), CEM (73; 95% CI: 63–81), MRI (69; 95% CI: 55–81), and HHUS (65; 95% CI: 46–80). **Conclusions:** The CEM, MRI, DBT, and HHUS demonstrate excellent performance in correctly identifying and classifying cancer lesions referred for diagnostic work-up, but HHUS, MRI, and CEM have a more limited ability to discriminate benign lesions than DBT and DM.

## Simple Summary

Radiologists often call back women whose screening mammograms show features that are suggestive of breast cancer for additional testing. Imaging modalities used for the additional testing of these women vary across countries and screening services; however, we do not know the imaging modality that best distinguishes between women who have and those who do not have breast cancer. We examined published studies to identify the best imaging modality that can be used for the testing of women who are recalled following mammography screening. We collected and analysed all the results in the studies published to date. Our analysis showed that contrast-enhanced mammography, digital breast tomosynthesis, magnetic resonance imaging, and ultrasound perform better than digital mammography in correctly identifying and classifying cancer lesions referred for additional testing; however, digital breast tomosynthesis and digital mammography perform better in correctly dismissing women who do not have cancer.

## 1. Introduction

Breast cancer remains the most common female malignancy and the foremost cause of cancer deaths in women worldwide. In 2020, breast cancer accounted for 1 in 4 new cancer cases and 1 in 6 cancer deaths [1,2]. Early detection of breast cancer has remained an important strategy for reducing breast cancer-related mortality. Mammography screening is the standard imaging tool for screening of asymptomatic women. Current evidence demonstrates that women who participate regularly in screening have a 41–60% reduced risk of death from breast cancer within 10–20 years [3,4], and a 25% reduced risk of advanced breast cancer [3]. The impact of screening mammography on breast cancer outcomes has led to the establishment of population-based screening programs in many countries.

To maximise the detection of breast cancer, radiologists often recall women whose mammograms show features that are suggestive of breast cancer to assessment clinics. The decision to recall women for assessment can be made by one radiologist in a single reading system or two radiologists in a double reading system, after at least two radiologists independently determine that a woman should be recalled [5,6]. The recall rates vary across screening programs and range from 9–14% for initial recall, but can be as low as 4% for subsequent recalls [7,8]. Only 1 in 10 women called back for assessment is eventually confirmed to have cancer [9]. Also, 6 in 10 women called back for additional testing, but confirmed to be cancer-free, have dense breasts even though they constitute approximately 30–40% of screened women [10].

Women recalled at screening undergo additional testing using different imaging modalities including magnified digital mammographic views (DM spot views), digital breast tomosynthesis, ultrasound, contrast-enhanced digital mammography, magnetic resonance imaging, and sometimes molecular breast imaging. In many countries, some of these imaging modalities are used concurrently, which could increase image interpretation time, cost of assessment, and patient radiation dose burden [11,12], particularly the 85–90% of women who receive a false-positive diagnosis [9]. There are also variations in the biopsy recommendation rates across imaging modalities [8] suggesting that an optimised imaging assessment of women recalled at screening has the potential to improve assessment outcomes, and could reduce unnecessary biopsies of benign lesions, over-testing, and associated risks and costs. However, imaging modalities or combinations of modalities that optimise diagnostic outcomes are poorly understood. As a result, there are variations in assessment protocols across screening services, which must be reduced to standardise care for women recalled at screening.

Studies have reported differences in the performance of different imaging modalities in cancer detection at screening and in the diagnostic setting including symptomatic women and those whose mammograms show changes that could indicate breast cancer [13,14,15,16,17,18,19,20]. Systematic reviews have established the comparative performances of these imaging modalities in the screening setting and as adjuncts to mammography; however, the comparative efficacy of these imaging modalities in women recalled at screening is poorly understood. The few published reviews either focus on symptomatic women or those at risk of cancer, assessed one imaging modality or comparator (digital mammography versus digital breast tomosynthesis or magnetic resonance imaging), and/or included studies combining populations with different indications including, but not limited, to screening, follow-up, preoperative staging of breast cancer, evaluation of palpable lumps, and work-up of abnormalities detected at screening [21,22]. Therefore, these reviews do not provide comprehensive evidence around the efficacy of imaging modalities used for the assessment of women recalled at screening. No review has examined the diagnostic efficacy of imaging modalities for asymptomatic women recalled at screening to establish optimised assessment pathways for women whose screening mammograms show features suspicious of breast cancer. Therefore, this paper aims to examine the diagnostic efficacy of different imaging modalities for the assessment of women recalled at screening.

## 2. Materials and Methods

***Search strategy:*** A systematic review of the literature was undertaken. The systematic review followed the recommendations of the Preferred Reporting Items for Systematic Reviews and MetaAnalyses (PRISMA). The protocol has not been registered. Eight databases were used to identify studies that assessed the efficacy of imaging modalities in the assessment of lesions recalled at screening until 10 August 2024. These databases were Medline via Ovid, Web of Science, Embase, Scopus, Science Direct, PubMed, CINAHL, and Global Health via Ovid. A Google Cross-search was conducted, and reference list of eligible articles are reviewed to identify articles missed on database search. Search terms were combined with Boolean operators and included the following: “Breast assessment” AND “Diagnostic Workup” OR “Mammography” AND “Digital Breast tomosynthesis” AND “contrast enhanced mammography” AND “Magnetic Resonance Imaging” AND “breast ultrasound”. A detailed search strategy is presented in Supplementary Table S1.

### 2.1. Inclusion and Exclusion Criteria

Studies were included if they met the following criteria: (1) examined the diagnostic performance of imaging modalities in the assessment of women recalled at screening; (2) provided data to establish true-positive, true-negative, false-positive, and false-negative results to enable the calculation of sensitivity, specificity, and accuracy; (3) used an established reference standard; (4) assessed imaging modalities (digital mammography [DM], digital breast tomosynthesis [DBT], handheld ultrasound [HHUS], contrast-enhanced mammography [CEM], or magnetic resonance imaging [MRI]) alone or in combination. Studies that compared modalities in diagnostic workup particularly if the dataset contained >80% screen-detected lesions were also considered eligible.

Exclusion criteria were studies that included the following: (1) assessed the screening outcomes of these imaging modalities; (2) were based on symptomatic women presenting for diagnostic imaging; (3) included a mixture of women with different indications such as screening, follow-up, preoperative staging of breast cancer, evaluation of palpable lumps, and work-up of abnormalities detected at screening; (4) women recalled at screening were <80% in the sample or the proportion of screen-recalled women in the sample could not be established; (5) assessed only tumour enhancement and the detection of contralateral cancers without providing information to establish sensitivity and specificity of the lesions detected at screening; (6) the specificity analysis was based on the detection and classification of additional lesions, not the index lesion for which the patient was assessed; (7) focused on detection of histologically proven cancers and reported only sensitivity values or pre-operative assessment of lesion characteristics. Review articles, opinion pieces, commentaries, editorials, letters to the editor, and conference proceedings/papers were also excluded along with articles not published in English language.

### 2.2. Study Selection

All articles identified were downloaded to an EndNote Library and two reviewers (JA and EE) independently screened each article for eligibility based on the inclusion criteria described above. Where there were disagreements, these were either resolved via discussion between the two reviewers or through a third reviewer who served as an arbitrator.

### 2.3. Quality Assessment

Two reviewers (EE and IH) independently performed quality assessment to identify the risk of bias based on the following four main domains in the Quality Assessment of Diagnostic Accuracy Studies–Comparative (QUADAS-C) checklist: patient selection, index test, reference standard, and flow and timing [23]. A weighted kappa was used to assess the agreement between the two assessors. Differences in study quality ratings were also resolved via discussions.

### 2.4. Data Extraction and Synthesis

Data were extracted independently by two of the authors (JA and EE) using a pre-designed template. Information such as study characteristics (author, year of publication, study design, country of study), population characteristics and adjustments (sample size, age, clinical history, breast density), image interpretation protocol, reference standard, radiologists’ expertise, intervention, and comparator imaging modalities (DM spot views ultrasound, DBT, CEM, or MRI), and the imaging assessment outcomes (diagnostic accuracy, sensitivity, specificity, and positive and negative predictive values) were extracted. Discrepancies were resolved via discussion.

### 2.5. Statistical Analysis

We estimated the pooled summary of sensitivity and specificity for each imaging modality based on the bivariate random effects model. For observer performance studies with multiple readers, the mean sensitivity and specificity scores of all readers were used for the meta-analysis. Studies that did not include normal cases and benign lesions or reported false-positive or false-negative rates were excluded from the meta-analysis. Heterogeneity between studies was assessed using the Higgins’ I^2^ statistics; values greater than 60% and ≥75% were considered substantial and considerable heterogeneity, respectively [24]. When substantial or considerable heterogeneity was detected, the threshold effect was investigated by visually inspecting the forest plots of sensitivity and specificity and the correlation coefficient estimates between sensitivity and the false-positive rate from the RevMan parameters. A correlation coefficient greater than 0.6 was considered a threshold effect. The differences between the 95% prediction estimates and 95% confidence eclipse in the hierarchical summary receiver operating characteristic curve (HSROC) were also examined for each imaging modality to further investigate the degree of heterogeneity across the studies. We also conducted a meta-regression by including study design covariates in bivariate model to assess if study design influenced the results and estimated the changes in sensitivity and specificity. Publication bias was assessed using Egger’s test. A two-sided *p* < 0.05 was considered statistically significant. All meta-analyses were conducted using the MetaDisc2 software package (Clinical Biostatistics Unit-Hospital Ramon Cajal, Madrid, Spain).

## 3. Results

**Figure 1:**
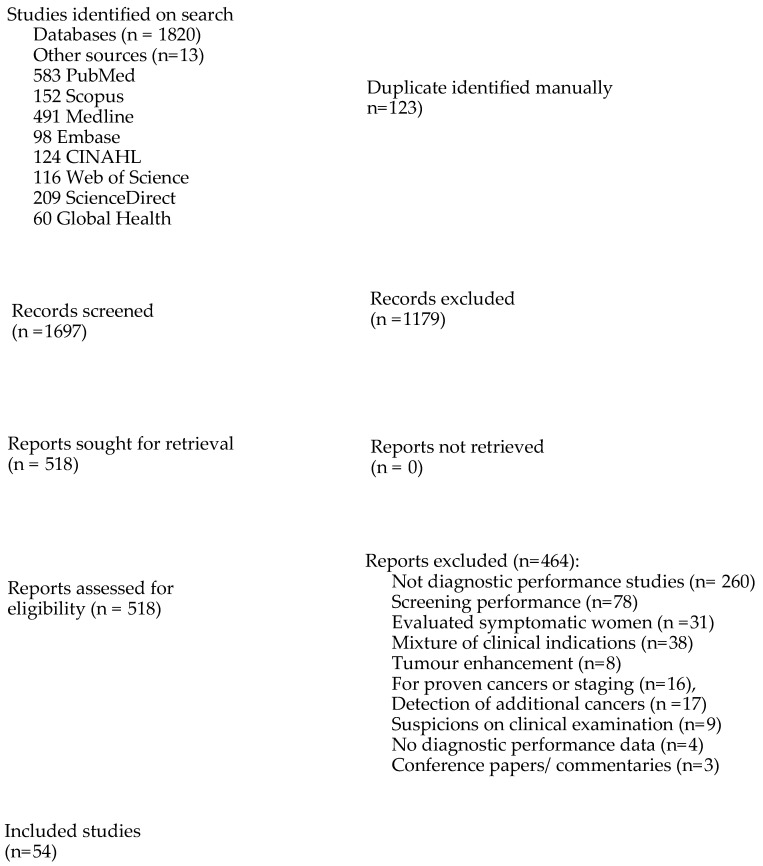
A PRISMA flowchart showing the excluded studies.

The literature search resulted in 1820 studies; 123 of these studies were duplicates. Following screening of title and abstract, 1179 were excluded, and 518 studies were considered appropriate for full-text review. After applying the inclusion and exclusion criteria, 464 of these 518 studies were excluded due to the following reasons: did not assess the diagnostic performance of imaging modalities (*n* = 260), assessed the performance of these imaging modalities for screening of asymptomatic women (*n* = 78), diagnostic evaluation of symptomatic women who had not undergone screening (*n* = 31), were for a mixture of different clinical indications (*n* = 38), assessed tumour enhancement only (*n* = 8), or focused on the detection of histologically proven cancers (*n* = 9) or staging of breast lesions (*n* = 7). Other reasons for exclusion were the following: detection of contralateral cancer or additional cancer not detected at screening (17), suspicious lesions at clinical breast examination (*n* = 9), did not provide diagnostic performance data (*n* = 4), or were conference papers and commentaries (*n* = 3). A total of 41 studies were considered eligible for inclusion. Thirteen additional studies were identified through Google search and reference lists of eligible articles. A PRISMA flowchart showing the excluded studies and reasons for exclusion is shown in Figure 1. Most of the studies reviewed included two or more imaging modalities.

A summary of the characteristics of the studies that assessed each of the imaging modalities including study design, number of lesions and mean ages of the women assessed, and publication bias is presented in Table 1.

Twenty-five studies assessed the performance of DM and compared DM with DBT in 22 studies [13,14,15,16,17,18,19,25,26,27,28,29,30,31,32,33,34,35,36,37,38,39] and with CEM in three studies [40,41,42]. Of these 25 studies, there were 12 prospective and 12 retrospective studies, with one study combining prospective and retrospective designs. Lesions assessed were mostly those rated 0, 3, 4, or 5 at screening. A total of 7801 lesions (malignant: *n* = 2833; benign/normal: *n* = 4968) were assessed and disease prevalence was 36%. Twenty-five studies assessed the performance of DBT [13,14,15,16,17,18,19,25,26,27,28,29,30,31,32,33,34,35,36,37,38,39] and compared DBT with US in three studies [43,44,45]. Of these 25 studies, there were 10 prospective and 14 retrospective studies, with one study combining prospective and retrospective designs. Lesions assessed were mostly BI-RADS 0, 3, 4, or 5. A total of 7191 lesions (malignant: *n* = 2387; benign/normal: *n* = 4804) were assessed and disease prevalence was 33%. Most of the studies involving DM and DBT were conducted in Egypt (*n* = 5), Germany (*n* = 4), Australia (*n* = 4), UK (*n* = 3), and the USA (*n* = 3). The mean ages of women assessed ranged from 44.2 to 61.5 and 84% of the studies included women of all breast densities. A summary of the characteristics of the 28 studies (DM vs. DBT: *n* = 22; DM vs. CEM: *n* = 3; DBT vs. HHUS: *n* = 3) that examined the performance of DM and DBT in recalled lesions is shown in Supplementary Table S2.

Ten studies assessed the performance of handheld ultrasound (HHUS) in recalled lesions, but nine studies reported the performance of HHUS alone. Three of these studies compared HHUS and automated breast volume ultrasound (ABVS) [46,47,48], four studies examined HHUS and DBT or DBT plus DM [43,44,45,49], and three with either DM + DBT or CEM [50,51,52]. Of these, there were two studies each in Australia, USA, and France, and one study each from the Netherlands, Germany, China, and Sweden. The mean ages of women assessed ranged from 43.1 to 60 and women of all breast densities were considered. Lesions assessed were mostly BI-RADS 3, 4, or 5. A total of 1972 lesions (malignant: *n* = 1019; benign/normal: *n* = 953) were assessed and disease prevalence was 52% (See Supplementary Table S3). A total of 16 studies examined the diagnostic performance of CEM in the work-up of lesions detected at screening [20,40,41,42,50,51,53,54,55,56,57,58,59,60,61,62]. Most of these studies (*n* = 15) were based on prospective research designs, and involved CEM alone [53,62], with DM or DBT plus DM or HHUS [40,41,42,50,51,54,55,57,58,59], and MRI in five studies [20,55,56,60,61]. Three of these studies were conducted in the Netherlands, and there were two studies each, in China, Italy, Egypt, Poland, and the USA, and one study each in France, Spain, and Austria (Supplementary Table S4). The mean ages of women assessed ranged from 48.5 to 58.4, and women of all breast densities were included. Lesions assessed were mostly BI-RADS 4 or 5. A total of 2975 lesions (malignant: *n* = 1710; benign/normal: *n* = 1265) were assessed and disease prevalence was 57%. Fourteen studies investigated the performance of MRI in recalled lesions [20,39,55,56,60,61,63,64,65,66,67,68,69]. Seven of these studies were prospective and seven studies used a retrospective research design. These studies either assessed MRI alone (*n* = 5) [63,64,65,66,68] or involved MRI with CEM (*n* = 5) [20,55,56,60,61] or HHUS (*n* = 2) [69,70] or DM (*n* = 2) [39,67]. These studies were conducted in the USA (*n* = 4), Egypt (*n* = 2), Italy (*n* = 2), Austria (*n* = 2), Poland (*n* = 1), China (*n* = 1), Germany (*n* = 1), and the Netherlands (*n* = 1). The mean ages of women assessed ranged from 48.5 to 56 and 93% of the studies included women of all breast densities as shown in Supplementary Table S5. Lesions assessed were mostly BI-RADS 0, 4, or 5. A total of 3629 lesions (malignant: *n* = 1286; benign/normal: *n* = 2343) were assessed and disease prevalence was 35%.

### 3.1. Quality Assessment

The quality assessment using QUADAS-C shows that in terms of patient selection, 51 studies had a low risk of bias [13,14,15,18,19,20,25,26,27,28,30,31,32,33,34,35,36,37,38,39,40,41,42,43,44,45,46,47,49,50,51,52,53,54,55,56,57,58,59,61,64,65,66,67,68,69], two studies demonstrated a high risk [17,63], and the risk of bias in one study was unclear bias [60]. For the index test, 37 studies demonstrated a low risk of bias [14,15,16,17,18,19,25,26,28,33,34,36,39,41,42], 12 studies had a high risk, and the risk of bias in five studies was unclear risk. Regarding the reference standard, 52 studies showed a low risk of bias [13,14,15,18,19,20,25,26,27,28,30,31,32,33,34,35,36,37,38,39,40,41,42,43,44,45,46,47,49,50,51,52,53,54,55,56,57,58,59,61,64,65,66,67,68,69], and only two had a high risk [17,39]. Finally, in the domain of flow and timing, 42 studies demonstrated a low risk of bias, three studies showed a high risk [30,32,70], and the risk of bias was unclear in nine studies [13,28,29,34,39,62,63,66,68] (see Supplementary Table S6). The overall inter-assessor agreement between the two assessors was almost perfect (Kw = 0.82).

### 3.2. Diagnostic Performance Evaluation

Pooled analysis of the performance of each imaging modalities: Table 2 summarises the pooled sensitivities and specificities of the five imaging modalities in the assessment of recalled lesions. The CEM demonstrated the highest sensitivity followed by MRI and both DBT and ultrasound. The DBT had the highest specificity followed by DM and CEM. There were moderate to considerable heterogeneity across studies, but meta-regression analysis demonstrated that the results were not influenced by the differences in study designs (*p* > 0.05 for all). Egger’s test did not show publication bias in studies assessing DM, DBT, HHUS, and CEM (*p* > 0.05 for all). Studies that examined MRI showed significant publication bias (*p* = 0.01).

**Figure 2:**
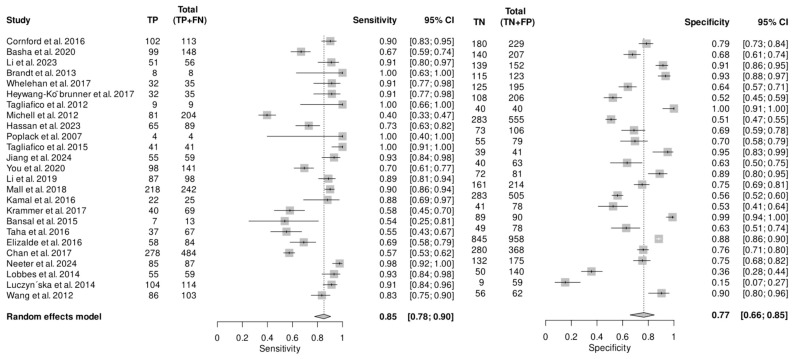
Forest plot illustrating the pooled sensitivity and specificity of DM in recalled lesions [12,13,14,15,16,17,18,19,25,26,27,28,29,31,32,33,34,35,36,37,38,39,40,41,42].

Of the 25 studies that assessed the performance of DM in recalled women, one study was excluded from the meta-analysis as it did not report false-negative and false-positive data [37]. The reported sensitivity varied from 40 (95% CI: 33–47) to 100 (95% CI: 40–100) and specificity ranged from 15 (95% CI: 7–27) to 100 (95% CI: 91–100). The pooled analysis showed that DM has sensitivity of 85 (95% CI: 78–90) and specificity 77 (95% CI: 66–85) in recalled lesions (Figure 2). There was considerable heterogeneity in sensitivity (I^2^ = 92%) and specificity (I^2^ = 96%), but no publication bias was found (*p* = 0.43). Data from these studies indicate that DM has lower diagnostic efficacy compared to other imaging modalities and that adding other assessment imaging modalities to DM improves diagnostic outcomes for women recalled at screening.

**Figure 3:**
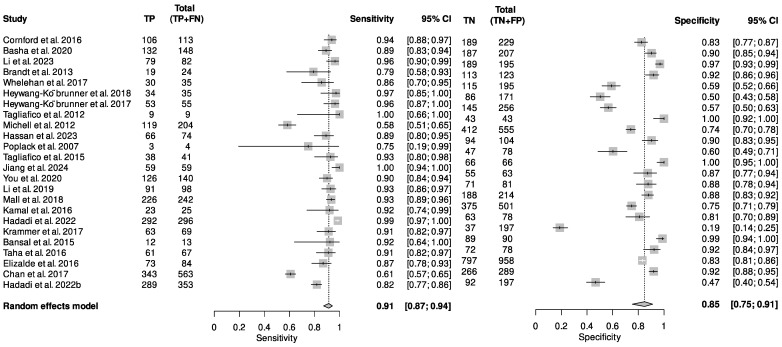
Forest plot illustrating the pooled sensitivity and specificity of DBT in recalled lesions [13,14,15,16,17,18,19,25,26,27,28,29,31,32,33,34,35,36,37,38,39,43,44,45].

A total of 24 of 25 studies provided sufficient data for meta-analysis of the performance of DBT in recalled women. The reported sensitivity values ranging from 58 (95% CI: 51–65%) to 100 (95% CI: 66–100) and specificity ranging from 19 (95% CI: 14–25%) to 100 (95% CI: 92–100). The pooled analysis showed that DBT has a sensitivity of 91 (95% CI: 87–94) and specificity 85 (95% CI: 75–91) in recalled lesions (Figure 3). The Higgin’s I^2^ statistics demonstrated considerable heterogeneity in sensitivity (I^2^ = 92%) and specificity (I^2^ = 96%), but no publication bias was detected (*p* = 0.69). Evidence from these studies show that DBT improves diagnostic efficacy in recalled lesions.

**Figure 4:**
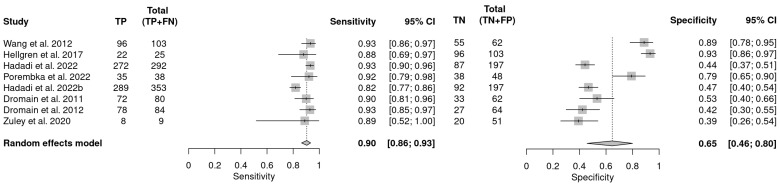
Forest plot illustrating the pooled sensitivity and specificity of HHUS in recalled lesions [43,44,46,48,49,50,51,52].

Of the 10 studies that assessed the performance of HHUS in recalled women, one combined HHUS and DBT one study did not provide sufficient data. Therefore, these studies were excluded from the meta-analysis. Of the remaining eight studies, sensitivity varied from 88 (95% CI: 69–97) to 94 (95% CI: 81–99) and specificity ranged from 39 (95% CI: 26–54) to 93 (95% CI: 86–97%). The pooled analysis showed that HHUS has sensitivity of 90 (95% CI: 86–93) and specificity 65 (95% CI: 46 –80) in recalled lesions (Figure 4). Heterogeneity was substantial in sensitivity (I^2^ = 72%) and considerable in specificity (I^2^ = 92%), but no publication bias was detected (*p* = 0.09). The findings from these studies suggest that HHUS is an effective imaging modality for the assessment of recalled lesions and has the potential to reduce the benign biopsy rates.

**Figure 5:**
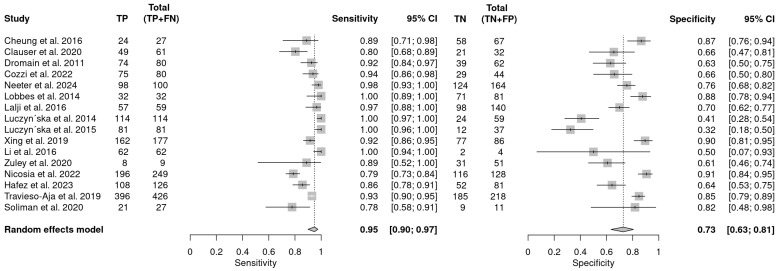
Forest plot illustrating the pooled sensitivity and specificity of CEM in recalled lesions [20,40,41,42,50,51,53,54,55,56,57,58,59,60,61,62].

Seventeen studies reported the performance of CEM in discriminating recalled lesions. Of these, the threshold for assessing performance or reporting of diagnostic performance was unclear in one study and was excluded from the meta-analysis. The remaining 16 studies reported sensitivity values ranging from 78 (95% CI: 58–91) to 100 (95% CI: 89 –100) and specificity ranging from 32 (95% CI: 18–50) to 91 (95% CI: 84–95). The pooled sensitivity was 95 (95% CI: 90–97%) and specificity was 73 (95% CI: 63–81) as shown in Figure 5. The I^2^ statistics demonstrated substantial heterogeneity in sensitivity (I^2^ = 72) and specificity (I^2^ = 88). No publication bias was detected (*p* = 0.72). The findings indicate that CEM is an efficient imaging modality for the assessment of recalled lesions.

**Figure 6:**
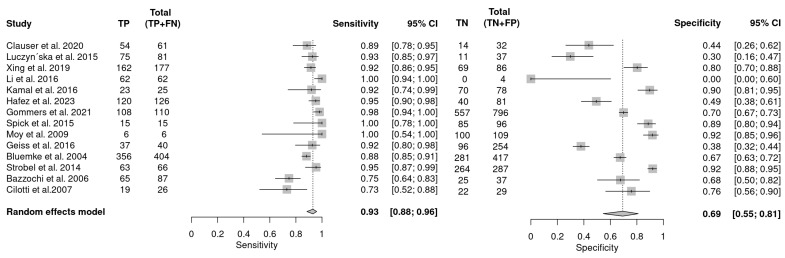
Forest plot illustrating the pooled sensitivity and specificity of MRI in recalled lesions [20,39,55,56,60,61,63,64,65,66,67,68,69,70].

In the 14 studies that met inclusion criteria, the sensitivity of MRI in recalled lesions varied from 73 (95% CI: 52–88) to 100 (95% CI: 54–100) and specificity ranged from 0 (95% CI: 0.00–0.6) to 92 (95% CI: 85–96). Figure 6 shows the forest plot of the MRI studies with a pooled sensitivity of 93 (95% CI: 88–96) and specificity of 69 (95% CI: 55–81). There was substantial to considerable heterogeneity (sensitivity; I^2^ = 68 and specificity; I^2^ = 95). Significant publication bias was observed (*p* = 0.01). The data produced indicate that MRI is highly sensitive and a useful imaging modality for resolving screen-recalled lesions, but is less efficient for resolving breast microcalcifications.

### 3.3. Comparative Performance of Imaging Modalities

For the 22 studies that assessed both DM and DBT, 21 studies provided results for comparison. Most of these studies (15/21) showed DBT to be superior to DM and that adding DBT to the assessment pathway improves diagnostic efficacy. Overall, the integrated sensitivity (88; 95% CI: 86–89) and specificity (79; 95% CI: 78–80) of DBT were higher than the sensitivity (71; 95% CI: 69–73) and specificity (72; 95% CI: 71–74) of DM. Similarly, the three studies that compared DM and CEM demonstrated higher sensitivity (99; 95% CI: 97–99) and specificity 72 (95% CI: 67–77) for CEM than DM (sensitivity: 95; 95% CI: 91–97 and specificity 51; 95% CI: 46–56). The limited data available show that CEM is superior or comparable to DM in recalled lesions. Only three studies compared DBT and HHUS and reported a higher integrated sensitivity (90; 95% CI: 87–92) but lower specificity (33; 95% CI: 28–38) for DBT compared to HHUS (sensitivity: 87; 95% CI: 84–90 and specificity: 45; 95% CI: 40–51). Data from these few studies indicate that DBT is superior to HHUS in detecting cancer lesions, but inferior or equivalent to HHUS in discriminating benign lesions. No study provided data to compare DBT alone with CEM or MRI. Five studies compared CEM and MRI, and the integrated analysis demonstrated comparable sensitivity between CEM and MRI (CEM: 93; 95% CI: 91–95 versus MRI: 91; 95% CI: 88–94), but lower specificity for CEM 56; 95% CI: 49–62) than MRI (68; 95% CI: 62–74). However, most of these studies (*n* = 4/5) considered CEM to have a better diagnostic performance based overall diagnostic accuracy.

## 4. Discussion

Pooled analysis of published evidence shows that at least 90% of cancer lesions referred for diagnostic workup can be identified and correctly classified by contrast-enhanced mammography, magnetic resonance imaging, digital breast tomosynthesis, and ultrasound, and that overall, these imaging modalities perform better than digital mammography in the assessment of women recalled from screening. However, the ability of these imaging modalities to correctly identify lesions that are not cancerous vary, with non-contrast X-ray imaging using digital breast tomosynthesis or digital mammography demonstrating better ability to correctly classify benign lesions referred for diagnostic work-up than handheld ultrasound, MRI, and CEM. The pooled specificity of DBT was 20%, 16%, and 12% higher than HHUS, MRI and CEM, respectively. When the analyses were limited to studies comparing imaging modalities on the same cohort of women, CEM consistently emerged the best imaging modality for accurately classifying cancer lesions, but its ability to exclude women without breast cancer was lower than digital mammography and MRI.

In the assessment clinic, radiologists have prior knowledge of the screening results of women recalled. It can be argued that recalling women based on mammography findings may bias the comparison of DM and other imaging modalities, and may have contributed to the lower sensitivity and specificity reported for DM across studies; whilst 68% of the studies that assessed the efficacy of DM in recalled women employed a prospective design, it was unclear whether the prospective nature was based on the comparator, which in most studies was DBT. Regardless, meta-regression analysis demonstrated no significant differences between prospective and retrospective studies in terms of sensitivity and specificity. Also, the prospective observer performance studies included in the analysis consistently demonstrated that DM was inferior to DBT, suggesting that recall based on mammography findings may have a negligible impact on the evidence around the role of DM in the assessment clinic. The higher sensitivity of CEM, MRI, DBT, and HHUS over DM can also be explained by the additional information provided by these imaging modalities. For example, the pseudo-3-dimensional images produced by DBT reduce breast tissue overlap and may allow radiologists to better discriminate lesions. Handheld ultrasound provides opportunities for radiologists and sonographers to interrogate the lesions in real-time and to assess the internal and external vascularity of the lesions as well as vascularity at the poles of the lesion [71]. Contrast enhancement and patterns of enhancement in CEM and MRI can also be used to determine whether a lesion is malignant or benign. However, some benign lesions produce enhancement on CEM and MRI or hypervascularity on ultrasound, which increase the potential for false-positive diagnosis; these enhancements and hypervascularity in benign lesions may explain the lower overall specificity reported for HHUS, MRI, and CEM [20,55,56,60,61]. Thus, enhancements in MRI and CEM, and vascularity in ultrasound can be strengths and drawbacks of these imaging modalities. Conversely, the lack of enhancement and abnormal enhancements due to contrast uptake in the normal parenchymal tissues in both CEM and MRI may lead to a false-negative diagnosis [72]. In addition, the risk of radiation exposure from CEM and adverse reactions to iodinated contrast media from both CEM and MRI are other factors to consider for women attending breast assessment clinics [73].

Overall, there were wide variations across studies in the diagnostic performance outcomes reported for each of the imaging modalities. These variations were more pronounced for specificity. The MRI demonstrated the widest variation from 0% to 92 followed by DM (15 to 100%) and DBT (19% to 100%). Surprisingly, HHUS, which is operator dependent and had the lowest pooled specificity demonstrated the least variation across studies. The lower specificity of HHUS, MRI, and CEM may be related to disease prevalence in studies assessing the diagnostic performance of these imaging modalities. The prevalence of cancer in MRI, CEM, and HHUS studies were 52%, 57%, and 33%, respectively; at higher disease prevalence, the specificity of a diagnostic test is reduced [74]. The wide variations in the specificity suggest that factors other than the imaging modalities or features of the lesions may have impacted diagnostic performance outcomes reported across studies. For example, it is well-established that subjective human variability leads to differences in imaging interpretation and that intrinsic human limitations affect the interpretation of imaging examinations [75,76]. Observer performance studies included in this review and other literature also demonstrate differences in diagnostic outcomes between radiologists, and these differences appear to be influenced by practice and workload characteristics [77,78]. Since the results presented in this meta-analysis represent the interpretations of different radiologists across different countries and practices, the impact of reader experience on the performance of each of these imaging modalities cannot be excluded. Other factors that may be responsible for the wide variations in performance outcomes across studies may be different recall thresholds for establishing the presence of cancer and differences in the distributions and characteristics of the lesions assessed. Whilst some studies considered lesions rated BI-RADS 4 and 5 as malignant, others considered BI-RADS 3, 4, and 5 [43,44,46,49,54,66] or 0, 4, and 5 [32,38,45,47,63]. Also, some studies included a mixture of masses, architectural distortions, calcifications, non-specific density lesions whereas other studies focused on calcifications or soft tissue lesions.

A useful imaging investigation should be able to detect or exclude a disease. Imaging guidelines also justify the appropriateness of an imaging investigation based on evidence of diagnostic impact, cost-effectiveness, and radiation dose. Such recommendations ensure the effective use of resources and maximise the benefits accrued from an imaging investigation. However, non-availability of appropriate imaging modalities and lack of confidence in the diagnosis from one imaging modality may lead to the use of the available imaging modality or multiple testing of women recalled at screening. Interestingly, evidence from the studies reviewed support the use of either CEM, MRI, DBT, or HHUS to correctly classify malignant lesions that were suspicious on DM; however, most of the studies show that the ability of these imaging modalities to correctly discriminate benign lesions is below 80%. However, the publication bias observed in MRI studies limits the strength and certainty of evidence for the use of MRI as an assessment tool. The findings suggest that imaging assessment alone may not be able to completely rule out cancer in all benign lesions identified at screening, and that biopsy and pathological analysis may still be required to dismiss suspicious screening mammography findings in some women recalled to assessment clinics.

There was a tendency for some studies to claim a prospective design even though retrospective data of mammograms appeared to have been used for the analysis. While some observer performance studies were classified as prospective by authors, others were identified as retrospective. Although meta-regression did not show any difference between these study designs, the parameters used for the analysis may have been biased by the authors’ original classification of their designs. To mitigate the risk of bias and ensure that the findings can be generalised, we focused on studies that used appropriate populations and reference standards to assess the diagnostic performance of these imaging modalities. Our comparative performance analysis was also based on studies that used the same reference standard to compare imaging modalities. However, the high risk or unclear bias in the index test and flow and timing in 32% and 22% of the studies, respectively, may have impacted on the results presented. For example, the lack of blinding of the radiologists who interpreted the assessment images containing the index lesions may have conferred advantages to CEM, MRI, DBT, and HHUS. Image interpretation involves search, perception, and decision-making. In clinical practice, many radiologists participate in the diagnostic work-up of their recalled lesions, have access to the screening images and results of these women, and are aware of the location of the lesions within the breast. This implies that they are not blind to the mammography results, and that the interpretation of assessment images may not involve detection tasks, but rather decision-making to either upgrade or downgrade the lesions previously identified on mammograms. There was moderate to considerable heterogeneity across studies. The heterogeneity and limitations within studies may have influenced the pooled results. The influence of these factors on the pooled analysis limits the dependence on the pooled results to establish the best imaging pathway for lesions recalled at screening. These limitations must be addressed in randomised controlled trials or through sufficiently powered observer performance studies to allow informed selection of the choice of modalities for screen-recalled women.

A limitation of the review was that only studies published in English language were considered. Due to language restrictions, some eligible articles may have been missed. However, most scientific findings are reported in English, and it has been shown that language restriction does not bias the findings of systematic reviews and meta-analyses [79]. We also included a few studies that did not specify whether the lesions included were recalled from screening with the assumption that the thresholds described for these lesions implied that they were recalled due to suspicious findings on mammograms. Conversely, this is the first review and meta-analysis to synthesise the literature on the efficacy of five different imaging modalities in the assessment of recalled lesions. Previous reviews focused on two or three imaging modalities and examined the performance of these modalities in a mixture of different clinical indications. Our review focused on problem-solving imaging for women recalled from screening and also highlighted the challenges in the literature that must be addressed to establish the best imaging pathway for screen-recalled women. Therefore, the findings should inform further research to support policies and practice around the assessment of women recalled at screening.

## 5. Conclusions

Incorporating either contrast-enhanced mammography, digital breast tomosynthesis, magnetic resonance imaging, or ultrasound in the assessment pathway of lesions recalled at screening improves sensitivity, but DBT and DM discriminate benign lesions better than CEM, MRI, and ultrasound.

## Figures and Tables

**Table 1:** A summary of the number and characteristics of studies that assessed each of the imaging modalities.

Modality	No. of Studies	Study Design	Mean Age Range	Number of Lesions	Assessment Threshold	Higgin’s I^2^	Egger’s Test
DM	25	P = 12 R = 12 P/R = 1	44.2–61.5	7801 Mal = 2833 B/N = 4968	0, 3, 4, or 5	Sen: 92% Spc: 96%	0.43
DBT	25	P = 10 R = 14 P/R = 1	44.2–61.5	7919 Mal = 2387 B/N = 4804	0, 3, 4, or 5	Sen: 92% Spc: 96%	0.69
HHUS	10	P = 7 R = 3 P/R = 0	43.1–60	1972 Mal = 1019 B/N = 953	3, 4, or 5	Sen: 72% Spc: 92%	0.09
CEM	16	P = 15 R = 1 P/R = 0	48.5–58.4	2975 Mal = 1710 B/N = 1265	4 or 5	Sen: 72% Spc: 88%	0.72
MRI	14	P = 7 R = 7 P/R = 0	48.5–56	3629 lesions Mal = 1286; B/N = 2343	0, 4, or 5	Sen: 68% Spc: 95%	0.01 *

DM: digital mammography; DBT: digital breast tomosynthesis; HHUS: hand-held ultrasound; CEM: contrast-enhanced mammography; MRI: magnetic resonance imaging; P = prospective; R: retrospective; P/R: prospective and retrospective; Mal: malignant; B/N: benign and normal; Sen: sensitivity; Spc: specificity; *: statistically significant.

**Table 2:** Summary of pooled performance (95% confidence interval) of imaging modalities for recalled lesions.

Imaging Modality	Sensitivity	Specificity
DM	85 (78–90)	77 (66–85)
DBT	91 (87–94)	85 (75–91)
HHUS	90 (86–93)	65 (46–80)
CEM	95 (90–97)	73 (63–81)
MRI	93 (88–96)	69 (55–81)

DM: digital mammography; DBT: digital breast tomosynthesis; HHUS: handheld ultrasound; CEM: contrast-enhanced mammography; MRI: magnetic resonance imaging.

## Data Availability

Not applicable.

## Supplementary Materials

The following supporting information can be downloaded at: https://www.mdpi.com/article/10.3390/cancers16203505/s1; Table S1: Search strategy for different databases. Table S2: Characteristics of studies assessing the role of DM and DBT in recalled lesions. Table S3: characteristics of the studies that examined the performance of ultrasound in the assessment of lesions recalled at screening. Table S4: characteristics of the studies that assessed the performance of contrast-enhanced mammography in screen-recalled lesions. Table S5: characteristics of the studies that assessed the performance of MRI in screen-recalled lesions. Table S6: Results of risk of bias assessment.
