# Supplementary material for: Diagnostic Efficacy of Five Different Imaging Modalities in the Assessment of Women Recalled at Breast Screening—A Systematic Review and Meta-Analysis
Source: Cancers (Basel). 2024 Oct 17;16(20):3505. doi: 10.3390/cancers16203505 (PMC11505902; doi:10.3390/cancers16203505)
Supplement: Supplementary file 1 [file cancers-16-03505-s001.zip › cancers-3213287-supplementary.pdf]

Supplementary Table S1: Search strategy for different databases

Medline via Ovid	"Assessment of screen-recalled" AND "Diagnostic Workup" OR "Mammography" AND "Digital Breast tomosynthesis" AND "contrast enhanced mammography and Magnetic Resonance imaging AND breast ultrasound in breast assessment"
Global Health via Ovid	Breast assessment OR Diagnostic Workup OR Mammography AND Digital Breast tomosynthesis AND contrast enhanced mammography and Magnetic Resonance imaging AND "breast ultrasound" in recalled lesions.
Web of Science	"Breast assessment" AND "Diagnostic Workup of breast lesions" OR "Mammography" AND "Digital Breast tomosynthesis" OR "contrast enhanced mammography and Magnetic Resonance imaging" AND "breast ultrasound" in breast assessment"
Embase	"Breast assessment" AND "Diagnostic Workup of breast lesions" OR "Mammography" AND "Digital Breast tomosynthesis" OR "contrast enhanced mammography and Magnetic Resonance imaging" AND "breast ultrasound" in breast assessment.
Scopus	"Breast assessment" AND "Diagnostic Workup of recalled lesions" OR "Mammography" AND "Digital Breast tomosynthesis" AND "contrast enhanced mammography and Magnetic Resonance imaging" AND "breast ultrasound in screen-recalled women"
Science Direct	"Mammography recall and Breast assessment" AND "Diagnostic Workup" OR "Mammography" AND "Digital Breast tomosynthesis" AND "contrast enhanced mammography and Magnetic Resonance imaging" AND "breast ultrasound"
PubMed	"Breast assessment" AND "Diagnostic Workup of recalled lesions" OR "Mammography" AND "Digital Breast tomosynthesis" AND "contrast enhanced mammography and Magnetic Resonance imaging" AND "breast ultrasound in screen-recalled women"
CINAHL	"Breast assessment" AND "Diagnostic Workup of recalled lesions" OR "Mammography" AND "Digital Breast tomosynthesis" AND "contrast enhanced mammography and Magnetic Resonance imaging" AND "breast ultrasound in screen-recalled women"

Supplementary Table S2: Characteristics of studies assessing the role of DM and DBT in recalled lesions

Author(year)	Country	Study design	No (Lesions)	Mean age	Breast density	Consecutive sampling	Per analysis	Index test/comparator	Reference standard	Assessment threshold	Conclusions
Cornford et al. 2016[13]	UK	Prospective and retrospective	342 (Malginant:113; benign 229)	NR	All	Yes	Lesion	DM; DBT;	Histopathology	M3,4,5	DBT and Spot views have similar performance
Basha et al. 2020[14]	Egypt	Prospective	296 (355)	46	All	No	Lesion	DBT, DBT+DM	Histopathology	BI-RADS 4	Adding DBT to DM improves diagnostic assessment
Heywangkbrunner 2017[38]	Germany	Prospective Observer performance	241 lesions	49.8	All	Yes	Lesion	DBT; DM	Histopathology	BI-RADS 3, 4, 5, and 0	DBT is equivalent to DM in assessment of recalled abnormalities
Li et al. 2023[25]	Australia	retrospective	210 (274)	NR	All	Yes	Lesion	DBT; DM	Histopathology	BI-RADS 3, 4, 5	DBT detects more lesions and may require additional procedures
Brandt et al. 2013[17]	USA	Retrospective	146 (158)	NR	All	Yes	Lesions	DBT	Imaging, histopathology, follow-up	BI-RADS 3, 4, 5	DBT can replace mammography for noncalcified soft-tissue lesions
Whelehan et al. 2017[26]	Germany	Retrospective	230	NR	All	Yes	Lesion	DBT; DM	Histopathology	BI-RADS 3, 4, 5, and 0	DBT demonstrates similar performance to DM in recalled abnormalities.
Lobbes et al. 2014[41]	Netherlands	Prospective	133	57.2	All	Yes	Patient	CEM; DM	Histopathology	4, 5	DM is inferior to CEM in women recalled at screening.

Tagliafico et al. 2012[35]	Italy	Prospective	52	51	All	Yes	Lesion	DBT; DM	Biopsy/histology	BI-RADS 0	The performance of DBT is equivalent to DM
Michell et al. 2012[27]	UK	Prospective	738 (759)		All	Yes	Lesion	DBT; DM	Surgical histology	M3/4, M5	DBT improves the diagnosis of soft-tissue mammographic abnormalities in women recalled at screening
Hassan et et al. 2023[33]	Egypt	prospective	148 (178)	44.2	All	Yes	Lesion	DBT; DM	Histology and follow-up	BI-RADS 4,5	DBT has better diagnostic performance than DM.
Poplack et al. 2007[32]	Lebanon	Prospective	99 (82)	57	All	Histology and follow-up	Lesion	DBT; DM	Histology	BI-RADS 3, 4, 5, 0	DBT can effectively supplement DM in assessment of screen-recalled women.
Tagliafico et al. 2015[18]	Italy	Prospective	107	49.3	All	Histology	Lesion	DBT; DM	Histology	R3, 4, 5	DBT may reduce biopsies of benign microcalcifications.
Jiang et al. 2024[34]	USA	Retrospective	122	47.7	All	Core needle biopsy/histology	Lesion	DBT; DM	Histology and follow-up	BI-RADS 3,4 5	DBT has better diagnostic efficacy than DM
You et al. 2020[28]	China	Prospective	212 (222)	NR	All	No	Lesion	DM; DBT+DM	Histology	BI-RADS 4	2D images from DBT improves diagnostic efficacy
Li et al. 2019[29]	China	Retrospective	305 (312)	49	All	No	Lesion	DBT; DM	Histology	BI-RADS 4B, 4C, and 5	DBT is superior to DM in discriminating benign lesions in dense breasts
Luczyn´ska et al. 2014[42]	Poland	Prospective	152 (173)	56	All	Yes	Lesion	CEM; DM	Histology	4, 5	DM is less sensitive than CEM in the work-up of lesions.

Mall et al. 2018[16]	Australia	Retrospective	144	NR	All	Yes	Patient	DBT; DM	Imaging, biopsy, follow-up	BI-RADS 4	DBT improves diagnostic efficacy in the breast cancer assessment
Kamal et al. 2016[39]	Egypt	Retrospective	98 (103)	48.6	BI-RADS C and D	No	Lesion	DBT, DM	Mixed	4	DBT and MRI are superior to DM in recalled lesions.
Neeter et al. 2024[40]	Netherla nds	Prospective	264	NR	All	Yes	Patient	CEM; DM	Histology	4, 5	DM is comparable to CEM for work-up of recalled women
Krammer et al. 2017[37]	Germany	Retrospective	66 (69)	61.5	C – D	No	Lesion	DBT; DM	Mixed	NR	DBT is superior to DM in lesion assessment and detection of additional cancers in dense breasts.
Bansal et al. 2015[31]	UK	Retrospective	103 (106)	53	B, C, D	Yes	Lesion	DBT+DM; DM	Histology and follow-up	M4	DBT increases diagnostic accuracy.
Taha et al. 2016[36]	Egypt	Prospective	132 (145)	59	All	No	Lesion	DM, DBT	Histology, cytology, follow-up	4	DBT improves diagnostic efficacy and lesion localisation
Elizalde et al. 2016[15]	Spain	Retrospective	1042	52	B, C, D	No	Patient	DBT+DM; DM	Mixed	3	DBT increases the sensitivity of assessment
Chan et al. 2017[19]	USA	Retrospective	134 (142)	46	All	yes	Lesion	DM; DBT	Mixed	4a	Radiologists distinguish lesions better in DBT than in DM
Ahmed et al. 2024[30]	Egypt	Retrospective	415	56.1	All	No	Lesion	DM; DBT; DM+DBT	Histopath ology	4	DBT+DM improve the classification of benign lesions
Hadadi et al. 2022[43]	Australia	Retrospective	482	59.3	All A – B vs C – D	No	Lesion	DBT, US	Histopath ology	RANZ CR 4,5	DBT has higher sensitivity, but lower specificity than ultrasound
Hadadi et al. 2022[44]	Australia	Retrospective	538	58.9	All	Yes	Lesion	DBT; US	Histopath ology	RANZ CR 4, 5	Ultrasound has greater potential to reduce the benign biopsy rate

Heywangkbrunner (2018)[45]	Germany	Observer performance	241	NR	All	Yes	Lesion	DBT; US	Histology and follow-up	BI-RADS 3, 4, 5, and 0	The performance of DBT is equivalent to US in assessment of recalled abnormalities

DM: digital mammography; CEM: contrast-enhanced mammography; US: ultrasound; MRI: magnetic resonance imaging.

Supplementary Table S3: characteristics of the studies that examined the performance of ultrasound in the assessment of lesions recalled at screening.

Author(year)	Country	Study design	No (Lesions)	Mean age	Breast density	Consecutive sampling	Per analysis	Index test/comparator	Reference standard	Assessment threshold	Conclusions
Den Dekker et al. 2024[47]	Netherlands	Prospective	523 (43)	NR	All	Yes	Lesion	HHUS; ABVS	Histology	BI-RADS 0	3D ultrasound is inferior to HHUS and/or DBT in diagnostic work-up and biopsy recommendation.
Wang et al. 2012[48]	China	Prospective	155 (165)	43.1	All	Yes	Lesion	ABVS, HHUS+DM	Histology	BI-RADS 3, 4, 5	ABVS has better sensitivity than mammography, but

											similar performance to US.
Hellgren at al. 2017[46]	Sweden	Prospective	113 (118)	55.6	All	Yes	Lesion	HHUS; ABVS	Histology, cytology	BI-RADS 3, 4, 5,	ABVS may be better than handheld US for the work-up of women recalled from screening mammography
Hadadi et al. 2022[43]	Australia	Retrospective	482 (492)	59.3	All	Yes	Lesion	DBT; US	Histopathology	BI-RADS 3, 4, 5	DBT detects more lesions and may require additional procedures
Porembka et al. 2022[49]	USA	Prospective	399 (430)	60	All	Yes	Lesions	US; DBT	Imaging, Histology, follow-up	BI-RADS 3, 4, 5	US is an effective tool for work-up of noncalcified masses recalled on screening tomosynthesis.
Hadadi et al. 2022[44]	Australia	Retrospective	538	58.9	All	Yes	Lesion	DBT; US	Histopathology	BI-RADS 3, 4, 5	Ultrasound has greater potential to reduce the biopsy of benign lesions than DBT
Dromain et al. 2012[52]	France	Prospective	122 (184)	57	All	Yes	Patient and Lesion	CEM; DM; US	Cytology and Histology	4,5	The addition of CEM in the diagnostic work-up improves diagnostic accuracy than USS
Heywangkbrunner (2018)[45]	Germany	Observer performance	241	NR	All	Yes	Lesion	US+DBT; US+DM	Histology and follow-up	BI-RADS 3, 4, 5, and 0	The performance of DBT is equivalent to US in assessment of recalled abnormalities

Dromain et al. 2011[51]	France	Prospective	120 (142)	56	All	Yes	Lesion	CEM; DM; DM+US	Cytology and Histology	4,5	CEM has better diagnostic accuracy than DM or DM+USS
Zuley et al. 2020[50]	USA	Retrospective	54 (60)	50	All	Yes	Lesion	CEM; DM+DBT; US	Histology	4	CEM reduces biopsy of benign lesions without affecting cancer detection.

Supplementary Table S4: characteristics of the studies that assessed the performance of contrast-enhanced mammography in screen-recalled lesions

Author(year)	Country	design	No. (Lesions)	Mean age	Breast density	Consecutive sampling	Per analysis	Index test/comparator	Reference standard	Assessment threshold	conclusions
Cheung et al. 2016[53]	China	Prospective	87 (94)	54	All	Yes	Lesion	CEM	Histology	BI-RADS 4	CEM adds value to assessment of non-mass breast microcalcification,
Clauser et al. 2020[61]	Austria	Prospective	80 (93)	54.3	All	Yes	Lesion	CEM; MRI	Histology	4, 5	CEM performs better and reduce false positive biopsies than MRI.
Dromain et al. 2011[51]	France	Prospective	120 (142)	56	All	Yes	Lesion	CEM; DM; DM+USS	Cytology and Histology	4,5	CEM has better diagnostic accuracy than DM or DM+USS
Lobbes et al. 2014[41]	Netherlands	Prospective	133	57.2	All	Yes	Patient	CEM; DM	Histology	4, 5	CEM improves diagnostic performance in women recalled at screening.
Lalji et al. 2016[57]	Netherlands	Prospective	199	58.4	All	Yes	Patient	CEM; DM	Histology	4,5	CEM has excellent problem-solving capabilities in women referred from screening.
Cozzi et al. 2022[54]	Italy	Prospective	220 (225)	56.6	All	Yes	Lesion	CEM; DM, DBT, USS	Histology	3,4,5	CEM provides optimal sensitivity and significantly reduces the benign biopsy rate
Neeter et a. 2024[40]	Netherlands	Prospective	264	NR	All	Yes	Patient	CEM; DM	Histology	4, 5	CEM is comparable to DM for work-up of recalled women but more efficient assessment pathway.

Soliman et al. 2020[58]	Egypt	Prospective	360	NR	All	Yes	Patient	CEM; DM+BUS	Histology	4,5	CEM performs better than DM+BUS in lesion detection and reducing benign biopsies
Travieso-Aja1. 2019[59]	Spain	Retrospective	465 (644)	51.5	All	Yes	Lesion	CEM; DM; DB+BUS	Histology	4, 5	CEM has a high diagnostic efficacy for assessment of breast lesions.
Luczyn´ska et al. 2014[42]	Poland	Prospective	152 (173)	56	All	Yes	Lesion	CEM; DM	Histology	4, 5	CEM is more sensitive and has better diagnostic accuracy in the work-up of lesions
Luczyn´ska et al. 2015[20]	Poland	Prospective	102 (118)	NR	All	Yes	Lesion	CEM; MRI	Histology	4, 5	CESM is potentially valuable in the diagnostic work-up of lesions
Xing et al. 2019[60]	China	Prospective	235 (263)	51	All	Yes	Lesions	CEM; MRI	Histology	4, 5	CEM has better diagnostic performance than MRI
Li et al. 2017[56]	USA	Retrospective	48 (66)	56	All	No	Lesion	CEM; MRI	Histology	4,5	CEM has equivalent sensitivity to MRI
Nicosia et al. 2022[62]	Italy	Prospective	321 (377)	51 (median)	All	Yes	Lesion	CEM	Histology	Malignancy score 2, 3	Absence of enhancement in CEM reduces the benign biopsy rate
Zuley et al. 2020[50]	USA	Retrospective	54 (60)	50	All	Yes	Lesion	CEM; DM+DBT; US	Histology	4	CEM reduces biopsy of benign lesions without affecting cancer detection.
Hafez et al. 2023[55]	Egypt	Retrospective	122 (126)	48.5	All	No	Lesion	CEM; MRI	Histology	4, 5	CEM has a higher specificity but lower sensitivity than MRI

DM: digital mammography; CEM: contrast-enhanced mammography; US: ultrasound; MRI: magnetic resonance imaging.

Supplementary Table S5: characteristics of the studies that assessed the performance of MRI in screen-recalled lesions

Author(year)	Country	design	No. (Lesions)	Mean age	Breast density	Consecutive sampling	Per analysis	Index test/comparator	Reference standard	Assessment threshold	conclusions
Clauser et al. 2020[61]	Austria	Prospective	80 (93)	54.3	All	Yes	Lesion	CEM; MRI	Histology	4, 5	CEM performs better and reduce false positive biopsies than MRI.
Luczyn´ska et al. 2015[20]	Poland	Prospective	102 (118)	NR	All	Yes	Lesion	CEM; MRI	Histology	4, 5	CEM has a higher negative predictive value and similar false positive rate to MRI. CEM is valuable in the diagnostic work-up of lesions
Xing et al. 2019[60]	China	Prospective	235 (263)	51	All	Yes	Lesion	CEM; MRI	Histology	4, 5	CEM has better diagnostic performance than MRI
Li et al. 2017[56]	USA	Retrospective	48 (66)	56	All	No	Lesion	CEM; MRI	Histology	4,5	CEM has equivalent sensitivity to MRI

Hafez et al. 2023[55]	Egypt	Retrospective	122 (126)	48.5	All	No	Lesion	CEM; MRI	Postoperative pathology	4, 5	CEM has a higher specificity but lower sensitivity than MRI
Gommers et al. 2021[63]	Netherlands	Retrospective	906	NR	All	Yes	Lesion	MRI	Histology	0, 3, 4, 5	MRI has potential to reduce further assessment of benign lesions
Spick et al. 2015[64]	Austria	Retrospective	111	51	All	Yes	Patient	MRI	Histology	0	MRI reliably excludes malignancy in BI-RADS 0 lesions
Moy et al. 2009[68]	USA	Retrospective	109	NR	All	No	Lesion	MRI	Histology	0	MRI is a useful tool for resolving inconclusive DM findings.
Giess et al. 20117[70]	USA	Retrospective	294	55	All	Yes	Lesion	MRI, US	Histology	0, 3	MRI has high sensitivity for inconclusive lesions.
Bluemke et al. 2004[67]	USA	Prospective	821	NR	All	Yes	Lesions	MRI; DM	Histology	4, 5	MRI has high sensitivity but moderate specificity
Strobel et al. 2015[69]	Germany	prospective	353	53.9	All	Yes	Lesion	MRI; US	Histology	4	MRI is a useful tool for work-up of BI-RADS 4 lesions
Bazzochi et al. 2006[65]	Italy	Prospective	112	NR	All	Yes	Lesion	MRI	Histology	5	MRI is not perfectly sensitive for microcalcifications
Cilotti et al.2007[66]	Italy	Prospective	55	56	All	Yes	Lesion	MRI	Histology	3, 4, 5	MRI has suboptimal performance in the evaluation of microcalcifications
Kamal et al. 2016[39]	Egypt	Retrospective	98 (103)	48.6	C and D	No	Lesion	MRI, DBT, DM	Mixed	4	MRI performs better than DM

DM: digital mammography; CEM: contrast-enhanced mammography; US: ultrasound; MRI: magnetic resonance imaging.

Supplementary Table S6: Results of risk of bias assessment

Author, year	Test	Risk of Bias (QUADAS-C)			
		P	I	R	FT
Cornford et al. 2016	DM; DBT	✓	X	✓	?
Basha et al. 2020	DM; DBT	✓	✓	✓	✓
Heywang-Köbrunner et al. 2017	DM; DBT	✓	✓	✓	✓
Li et al. 2023	DM; DBT	✓	✓	✓	✓

Brandt et al. 2013	DM; DBT	X	✓	X	✓
Whelehan et al. 2017	DM; DBT	✓	✓	✓	✓
Lobbes et al. 2014	DM; CEM	✓	✓	✓	✓
Tagliafico et al 2012	DM; DBT	✓	✓	✓	✓
Michell et al. 2012	Film-screen; DM; DBT	✓	X	✓	✓
Hassan et al. 2023	DM; DBT	✓	✓	✓	✓
Poplack et al. 2007	DM; DBT	✓	X	✓	X
Tagliafico et al 2015	DM; DBT	✓	✓	✓	✓
Jiang et al. 2024	DM; DBT	✓	✓	✓	?
You et al. 2020	DM; DBT; SM	✓	✓	✓	?
Li et al. 2019	DM; DBT	✓	✓	✓	?
Luczyńska et al. 2014	DM; CEM	✓	✓	✓	✓
Mall et al. 2018	DM; DBT	✓	✓	✓	✓
Kamal et al. 2016	DM, DBT and MRI	✓	✓	X	?
Neeter et al. 2024	DM; CEM	✓	X	✓	✓
Krammer et al. 2017	DM; DBT	✓	✓	✓	✓
Bansal and Young, 2015	DM; DBT	✓	X	✓	✓
Taha et al. 2016	DM; DBT	✓	✓	✓	✓
Elizalde et al. 2016	DM; DBT; HHUS	✓	X	✓	✓
Chan et al. 2017	DM; DBT	✓	✓	✓	✓
Ahmed et al. 2024	DM; DBT	✓	✓	✓	X
Hadadi et al. 2022a	DM; DBT; HHUS	✓	X	✓	✓
Hadadi et al. 2022b	DM; DBT; US	✓	X	✓	✓
Heywang-Köbrunner et al. 2018	DM; DBT; US	✓	✓	✓	✓

den Dekker et al. 2024	DM; DBT; HHUS	✓	X	✓	✓
Wang et al. 2012	DM; HHUS; ABVS	✓	X	✓	✓
Hellgren et al. 2017	DM; HHUS; 3D ABUS	✓	✓	✓	✓
Porembka et al 2022	DM; HHUS	✓	✓	✓	✓
Dromain et al 2012	DM; CEM; HHUS	✓	✓	✓	✓
Dromain et al 2011	DM; CEM	✓	✓	✓	✓
Zuley et al 2019	DM; DBT; HHUS; CEM	✓	✓	✓	✓
Cheung et al 2016	DM; CEDM	✓	✓	✓	✓
Clauser et al. 2020	CEM; MRI	✓	✓	✓	✓
Lalji et al. 2016	DM; CEM	✓	✓	✓	✓
Cozzi et al. 2022	DM; CEM	✓	✓	✓	✓
Soliman et al 2020	DM; CEM HHUS;	✓	✓	✓	✓
Travieso-Aja et al.	DM; CEM HHUS;	✓	✓	✓	✓
Łuczyńska et al 2015	DM; CESM MRI;	✓	✓	✓	✓
Xing et al .2019	DM; CEM; MRI	?	✓	✓	✓
Li et al. 2017	DM; CEM; MRI	✓	✓	✓	✓
Nicosia et al. 2022	DM; CEM	✓	?	✓	?

Hafez et al 2023	DM; CEM; MRI	✓	✓	✓	✓
Gommers et al. 2021	DM; MRI	X	?	✓	?
Spick et al. 2015	DM; MRI	✓	✓	✓	✓
Moy et al. 2009	DM; MRI	✓	?	✓	?
Giess et al. 2017	DM; MRI	✓	?	✓	x
Bluemke et al. 2004	DM; MRI	✓	x	✓	✓
Strobel et al. 2015	DM; HHUS; MRI	✓	✓	✓	✓
Bazzocchi et al. 2006	DM; MRI	✓	x	✓	✓
Cilotti et al. 2007	DM; MRI	✓	?	✓	?

FT: Flow and Timing; I: Index Test; P: Patient Selection; R: Reference Standard; _✓: low risk; X: high risk; ?: unclear risk; DM: digital mammography; CEM: contrast-enhanced mammography; HHUS: handheld ultrasound; MRI: magnetic resonance imaging; ABVS: automated breast volume ultrasound.
